# Investigation of Hole-Transfer Dynamics through Simple EL De-Convolution in Non-Fullerene Organic Solar Cells

**DOI:** 10.3390/polym15204042

**Published:** 2023-10-10

**Authors:** Dongchan Lee, Do Hui Kim, Chang-Mok Oh, Sujung Park, Narra Vamsi Krishna, Febrian Tri Adhi Wibowo, In-Wook Hwang, Sung-Yeon Jang, Shinuk Cho

**Affiliations:** 1 Department of Semiconductor Physics and EHSRC, University of Ulsan, Ulsan 44610, Republic of Korea; 2 Advanced Photonics Research Institute, Gwangju Institute of Science and Technology, Gwangju 61005, Republic of Koreahwangiw@gist.ac.kr (I.-W.H.); 3 Department of Energy Engineering, School of Energy and Chemical Engineering, Ulsan National Institute of Science and Technology (UNIST), Ulsan 44919, Republic of Koreasyjang@unist.ac.kr (S.-Y.J.)

**Keywords:** organic solar cells, non-fullerene acceptor, charge transfer state, hole transport, energy loss

## Abstract

In conventional fullerene-based organic photovoltaics (OPVs), in which the excited electrons from the donor are transferred to the acceptor, the electron charge transfer state (*^e^E*_CT_) that electrons pass through has a great influence on the device’s performance. In a bulk-heterojunction (BHJ) system based on a low bandgap non-fullerene acceptor (NFA), however, a hole charge transfer state (*^h^E*_CT_) from the acceptor to the donor has a greater influence on the device’s performance. The accurate determination of *^h^E*_CT_ is essential for achieving further enhancement in the performance of non-fullerene organic solar cells. However, the discovery of a method to determine the exact *^h^E*_CT_ remains an open challenge. Here, we suggest a simple method to determine the exact *^h^E*_CT_ level via deconvolution of the EL spectrum of the BHJ blend (EL_B_). To generalize, we have applied our EL_B_ deconvolution method to nine different BHJ systems consisting of the combination of three donor polymers (PM6, PBDTTPD-HT, PTB7-Th) and three NFAs (Y6, IDIC, IEICO-4F). Under the conditions that (i) absorption of the donor and acceptor are separated sufficiently, and (ii) the onset part of the external quantum efficiency (EQE) is formed solely by the contribution of the acceptor only, EL_B_ can be deconvoluted into the contribution of the singlet recombination of the acceptor and the radiative recombination via *^h^E*_CT_. Through the deconvolution of EL_B_, we have clearly decided which part of the broad EL_B_ spectrum should be used to apply the Marcus theory. Accurate determination of *^h^E*_CT_ is expected to be of great help in fine-tuning the energy level of donor polymers and NFAs by understanding the charge transfer mechanism clearly.

## 1. Introduction

Organic photovoltaic cells (OPVs) based on the bulk-heterojunction (BHJ) blend of semiconducting polymers and small molecule acceptors are considered the most promising candidate as a future mobile energy source due to their advantages, including light weight, flexibility, and low-cost fabrication process [1,2,3,4,5]. The power conversion efficiency (PCE) of single-junction polymer solar cells has steadily increased and recently exceeded 18%. This was due to the introduction of non-fullerene acceptors (NFAs) [6,7,8]. The major advantage of NFAs is their relatively low energy loss, which can be as low as 0.4–0.5 eV [9,10]. It is generally believed that this energy loss is associated with the small charge transfer offset required to drive charge separation. In other words, charge separation efficiency is directly influenced by charge transfer offsets. Therefore, finding accurate information about the charge transfer (CT) state is essential for improving photovoltaic performance. 

**Figure 1:**
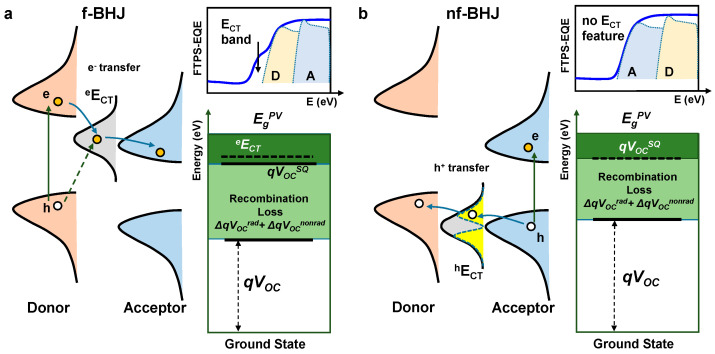
Difference between fullerene-based OSCs and non-fullerene-based OSCs near EQE onset (flat-band *V*_OC_ condition). (**a**) Electrons are initially excited at donor polymer and then transferred to acceptor in f-BHJ OSCs. *E*_CT_ level is visible in sensitive FTPS-EQE measurement. (**b**) Electrons are initially excited at acceptor near EQE onset. Holes are transferred to donor in nf-BHJ OSCs. *E*_CT_ level is invisible even in sensitive FTPS-EQE measurement.

In the BHJ system based on a fullerene acceptor (f-BHJ), the electron transfer state (*^e^E*_CT_) was determined by Marcus theory, which is based on the mirror image relationship between the photovoltaic external quantum efficiency (EQE_PV_) and electroluminescence (EL) emission [11,12]. All the charges that contribute to the onset part of the EQE_PV_ are initially excited at the donor site, and then electrons are transferred to the acceptor through the *^e^E*_CT_. Thus, if the EQE_PV_ is measured with a very sensitive tool such as Fourier transform photocurrent spectroscopy EQE (FTPS-EQE), it is expected that the CT-bands are visible in the low-energy part of the EQE_PV_ (see Figure 1a) [13,14]. In this framework, *^e^E*_CT_ was extracted by fitting the CT-band from the EQE_PV_ and projecting it on the EL spectrum (Figure S1 and Table S1). In the BHJ system based on a low bandgap non-fullerene acceptor (nf-BHJ), however, the electron density excited in the acceptor itself is already high, and the low-energy onset part of EQE_PV_ is also formed only by the contribution of the acceptor. Therefore, *^e^E*_CT_ created near the lowest unoccupied molecular orbital (LUMO) levels of donor and acceptor has little influence on the device performance, and there is no CT-band feature in the EQE_PV_ onset (see Figure 1b) [13]. Rather, in this non-fullerene acceptor system, the energy offset associated with the hole transfer occurring on the highest occupied molecular orbital (HOMO) side of the donor and acceptor is more important. Accordingly, many previous studies to date have discussed the hole transfer on the HOMO side rather than electron transfer on the LUMO side [15,16,17,18,19]. Of course, there may be a change in photocurrent due to the influence of *^e^E*_CT_ in operation under the saturation mode, but the influence of *^e^E*_CT_ is negligible because there are no electrons transferring from the donor in the *V*_OC_ formation near the EQE onset we consider.

A study by J. Zhang et al. [20] showed that the minimum HOMO offset needed to achieve the most efficient exciton dissociation and photovoltaic performance was ~40 meV, and the method for relative comparison of HOMO offset energy was demonstrated by Y. Xie et al. [21]. However, the former was just an estimate of the minimum value obtained through a simple comparison of the HOMO level difference and photovoltaic performance [20], and the latter was a relative comparison of estimated values obtained by determining EQE_EL_ difference [21]. Thus, the discovery of a method to determine hole transport energy offsets by accurately measuring the *E*_CT_ on the HOMO side (*^h^E*_CT_) remains an open challenge. In recent research by J. Wu et al., it was pointed out that the key factor of the high efficiency of the PM6:Y6 system was because of the low energetic disorder due to the low energy offset [22]. Therefore, the accurate determination of *^h^E*_CT_ is essential for achieving further enhancements in the non-fullerene organic solar cell since it can be a guideline for the design of a higher-performing novel material. 

In this work, we suggest a simple method to determine the exact *^h^E*_CT_ level via deconvolution of the EL spectrum of the BHJ blend (EL_B_). In general, the onset part of the EQE of the BHJ blend with NFA is formed solely by the contribution of the acceptor itself. In an EL measurement, electrons are injected directly into the acceptor LUMO, and holes are initially injected into the HOMO of the donor and then transferred to the acceptor through *^h^E*_CT_. Thus, EL_B_ has constraints on both the singlet recombination of the acceptor and the radiative recombination via *^h^E*_CT_. Therefore, by deconvolution of EL_B_, it is possible to extract out the part of CT-band contribution. Through this, we can clearly decide which part of the broad EL_B_ spectrum should be used to apply the Marcus theory. To generalize, we have applied our EL_B_ deconvolution method to nine different BHJ systems consisting of the combination of three donor polymers (PM6, PBDTTPD-HT, PTB7-Th) and three NFAs (Y6, IDIC, IEICO-4F).

## 2. Materials and Methods

### 2.1. Device Fabrication

All organic solar cell devices were fabricated with an inverted structure of ITO/ZnO/BHJ/MoO_3_/Ag. Patterned indium tin oxide (ITO) on glass was was purchased from AMG (Seoul, Republic of Korea). It was cleaned in an ultrasonic bath of detergent, acetone (99.9%), and isopropyl alcohol (IPA, 99.9%) for 10 min each and UV-ozone treated for 60 min. A 0.75 M ZnO sol-gel was prepared with 3.28 g of zinc acetate dihydrate (Zn(CH_3_COO)_2_∙2H_2_O, 98%), 1 mL of ethanolamine (NH_2_CH_2_CH_2_OH, 99.5%), and 20 mL of 2-methoxyethanol (CH_3_OCH_2_CH_2_OH, 99.9%). The ZnO sol-gel was spin-coated on ITO glass at 5000 rpm for 30 s under ambient conditions and thermally annealed at 200 °C. The employed BHJs are as follows: PM6: Y6 (D:A = 1:1.2, 16.5 mg/mL in chloroform (CF, 99%) with 0.5 *v*/*v*% of chloronaphthalene (CN, ≥90%) additive), PM6: IDIC (D:A = 1:1, 18 mg/mL in CF with 0.5 *v*/*v*% 1,8-diiodooctane (DIO, 98%) additive), PM6: IEICO-4F (D:A = 1:1.5, 18 mg/mL in CF with 0.5 *v*/*v*% of CN additive), PBDTTPD-HT: Y6 (D:A = 1:1.4, 20 mg/mL in CF with 0.5 *v*/*v*% of DIO additive), PBDTTPD-HT: IDIC (D:A = 1:1.4, 18 mg/mL in CF with 0.8 *v*/*v*% of DIO additive), PBDTTPD-HT: IEICO-4F (D:A = 1:1.5, 20 mg/mL in CF with 0.5 *v*/*v*% of CN additive), PTB7-Th: Y6 (D:A = 1:1.5, 20 mg/mL in CF with 0.5 *v*/*v*% of CN additive), PTB7-Th: IDIC (D:A = 1:1.5, 20 mg/mL in CF with 0.5 *v*/*v*% of DIO additive), and PTB7-Th: IEICO-4F (D:A = 1:1.5, 20 mg/mL in CF with 3 *v*/*v*% of CN additive).The BHJs were spin-coated at 5000 rpm for 30 s on the ZnO layer and moved to a vacuum chamber. The hole-transport layer (MoO_3_, 99.9%, 5.5 nm) and metal electrode (Ag, 99.99%, 100 nm) were deposited via thermal evaporation under a vacuum of 2 × 10^−6^ Torr. All materials except donor and acceptor materials were purchased from Sigma Aldrich (St. Louis, MO, USA). Detailed information on PM6, PBDTTPD-HT, and Y6 can be found in our previous works [23,24]. PTB7-Th, IDIC, and IEICO-4F were procured from 1-Material Inc. (Dorval, Quebec, Canada). The device area was 0.13 cm^2^.

### 2.2. Device Characterization

The power conversion efficiencies of the devices were measured by current density-voltage (*J-V*) curves using a Keithley 2401 (Keithley instruments, Cleveland, OH, USA) source measurement unit under AM 1.5 G (100 mW/cm^2^) illumination from a solar simulator (McScience, Suwon, Republic of Korea). The simulated light intensity was calibrated using a standard Si solar cell. External quantum efficiency (EQE) was measured using a solar cell spectral response/QE/IPCE (IQE-200B, Newport Co., Irvine, CA, USA). The light intensity at each wavelength was calibrated using a standard Si solar cell. 

The absorption spectra of each BHJ layer were measured by using a UV-Vis spectrophotometer (Cary 5000, Agilent Technologies Inc., Santa Clara, CA, USA). Electroluminescence (EL) signals passed through the integral sphere and were recorded by using a high-sensitivity spectrophotometer (MAYA 2000 Pro, Ocean Insight Inc., Orlando, FL, USA). The wavelength of the PL excitation source was 632 nm. The integral sphere and spectrophotometer were calibrated to collect the radiated photon flux from the device by using a standard halogen calibration light source (HL-3-plus-INT-CAL, Ocean Insight Inc., FL, USA).

Fourier transform photocurrent spectroscopy (FTPS) was used to perform measurements in an FTPS setup built in-house, which consisted of a Fourier-transform infrared spectrometer (INVENIO-R, Bruker Co., Billerica, MA, USA) equipped with quartz beam splitter. The photocurrent produced by the solar cell under illumination was amplified using a low-noise preamplifier (SR570, Stanford Research Systems, Sunnyvale, CA, USA) and fed back into the external detector port of the FTIR.

The steady-state photoluminescence (PL) spectra were obtained using a multichannel spectrophotometer (QEPro, Ocean Insight Inc., FL, USA). In PL measurements, acceptors and BHJs were excited from active layer side using a continuous wave diode laser (Nd-YAG tuned at 532 nm). To calculate the hole-transfer yields, PL spectra were divided by the absorption intensity at the 532 nm. Femtosecond transient absorption (TA) spectra were obtained using a homemade TA measurement system comprising a femtosecond Ti:sapphire regenerative amplifier system (Hurricane, Spectra-Physics Inc., Milpitas, CA, USA) with a 1 kHz repetition rate, an optical parametric amplifier (OPA-800CF, Spectra-Physics Inc., CA, USA), and multichannel spectrometers (QEPro and NIRQUEST, Ocean Insight Inc., FL, USA). The pump pulse wavelength was 630 nm with a power density of ~1 µJ/cm^2^.

## 3. Result and Discussion

### 3.1. Y6 Acceptor Based nf-BHJ Systems

#### 3.1.1. Background of *^h^E*_CT_ Determination

**Figure 2:**
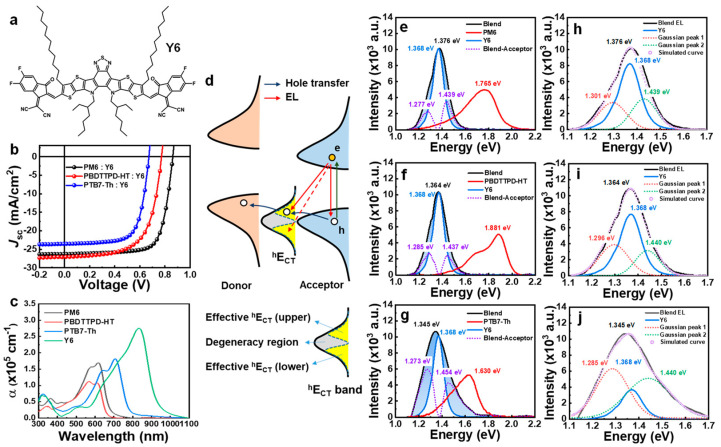
EL deconvolution analysis of the BHJ systems based on the Y6 acceptor. (**a**) Chemical structure of Y6 acceptor. (**b**) *J-V* characteristics of BHJ OPVs fabricated using Y6 acceptor. (**c**) Absorption coefficient spectra of polymer donors and Y6. (**d**) The schematic diagram of hole transfer in PV mode and charge recombination in EL mode. In EL mode, radiative recombination can occur in three ways: (i) recombination with acceptor singlet (mono-molecular recombination), (ii) recombination with holes at upper effective *^h^E*_CT_, or (iii) recombination with holes at lower effective *^h^E*_CT_. (**e**–**g**) EL spectra of BHJ blend, donor only, and acceptor only; shaded blue area (EL_B_-EL_A_) represents the EL contribution by *^h^E*_CT_. (**h**–**j**) Simulation of EL spectrum using Gaussian function and EL_A_.

We first analyzed the BHJ systems based on the representative non-fullerene acceptor Y6 (Figure 2a) with three donor polymers: PM6, PBDTTPD-HT, and PTB7-Th (Figure S2). Figure 2b shows the current density (*J*)–voltage (*V*) characteristics of OPVs. The parameters related to the performance of these OPVs are listed in Table S2. The acceptor Y6 has a lower bandgap and higher absorption coefficient than those of the three donors we used, as shown in Figure 2c. Thus, in the low-energy onset part of EQE spectra, there is no photocurrent contribution from the donor. All the contributions originated from Y6 only (Figure S4), which means that all photoexcited electrons are created in the acceptor and then extracted to the cathode directly. In the case of holes, they would be transferred to the donor through the *^h^E*_CT_ level and then extracted to the anode electrode (Figure 2d). Therefore, as similar to the FTPS-EQE onset of the f-BHJ system in which electrons generated from the donor are transferred to the acceptor has the information *^e^E*_CT_, the FTPS-EQE onset of nf-BHJ system will have information on *^h^E*_CT_. In the case of the f-BHJ system, since the ^*e*^*E*_CT_ bands are quite visible in the low-energy part of the FTPS-EQE spectrum, after determining the reorganization energy (*λ*) by fitting the *^e^E*_CT_ signal of the EQE, the fitting can be applied to the EL spectrum (Figure S1). However, in the case of the nf-BHJ systems, since there is no CT band feature in FTPS-EQE onset, we designed a way to secure the information of the *^h^E*_CT_ band from the EL spectrum and reflect it to FTPS-EQE. 

Under the EL measurement mode, the electrons are directly injected into the LUMO of the acceptor, while the holes are injected into the HOMO of the donor and transferred to the acceptor through *^h^E*_CT_. Thus, the hole injection can be hindered because the HOMO offset acts as a potential barrier. In EL_B_, of course, the EL emission from the singlet of the acceptor will be dominant. However, since there is the possibility of radiative recombination between electrons at LUMO of acceptor and holes at the *^h^E*_CT_, both radiative charge recombination from singlet excitons and *^h^E*_CT_ states are superimposed in the EL_B_ spectrum. Our first goal is to extract the contribution of the *^h^E*_CT_ in EL_B_ to apply the Marcus theory from EL to FTPS-EQE. To achieve this, the exact position of *^h^E*_CT_ emission should be addressed. To estimate the underlying contribution from *^h^E*_CT_, we have simply subtracted the acceptor EL (EL_A_) spectrum from EL_B_. Figure 2e–g shows the difference in the EL spectrum obtained from PM6:Y6, PBDTTPD-HT:Y6, and PTB7-Th:Y6, respectively. The difference in the EL spectrum (shaded blue area) represents the EL contribution from *^h^E*_CT_.

#### 3.1.2. EL Deconvolution

To determine the exact peak position, Gaussian fittings for EL_B_ were performed based on the information about the components obtained from EL_B_-EL_A_. In the case of PM6:Y6 EL_B_ (Figure 2h), the spectrum was deconvoluted into three components. The peak in the center (1.372 eV) is obviously the singlet radiative recombination between the LUMO and HOMO of the acceptor. The peaks on both the low- and high-energy side are caused by the effective *^h^E*_CT_ band. It is likely that *^h^E*_CT_ does not exist at a finite level. It has a broad distribution near the acceptor’s HOMO. The *^h^E*_CT_ levels near the acceptor’s HOMO are like a degenerate state, so it will be indistinguishable. Therefore, the *^h^E*_CT_ band can be divided into three regions (Figure 2d). What we are interested in is the energy range that is lower than the acceptor’s HOMO (higher effective *^h^E*_CT_), which affects energy loss. The *^h^E*_CT_ levels in the energy region that is higher than the acceptor’s HOMO (lower effective *^h^E*_CT_) will be excluded from the analysis because this part has no effect on the energy loss of the solar cells. Moreover, there is some overlap with the tail of the donor’s EL (EL_D_). The results of the same Gaussian fittings for PBDTTPD-HT and PTB7-Th are shown in Figure 2i,j, respectively. In the case of the PBDTTPD-HT:Y6 blend, EL spectrum features are similar to the PM6:Y6 blend due to the similar energy band configurations. However, in the case of the PTB7-Th:Y6 system, it was determined that the contribution of singlet excitons was lower than that of *^h^E*_CT_. Simply put, this means that the energy loss caused by *^h^E*_CT_ is greater in the PTB7-Th:Y6 blend.

#### 3.1.3. Marcus Theory and Energy Loss Model

**Figure 3:**
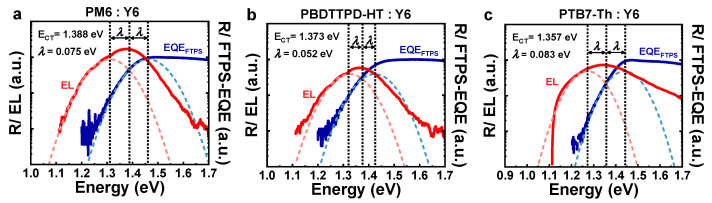
Δ*^h^E*_CT_ was determined based on the Marcus theory for (**a**) PM6:Y6, (**b**) PBDTTPD-HT:Y6, and (**c**) PTB7-Th:Y6 BHJs. The position of *^h^E*_CT_ emission was obtained from EL_B_ deconvolution and then fit to FTPS-EQE onset part. The CT absorption (blue dotted line) and CT emission (red dotted line) were fitted using Gaussian function.

Now, we can apply the Marcus theory (Appendix A) to determine the exact Δ*^h^E*_CT_ (=*E*_g_^PV^ − *^h^E*_CT_), where *E*_g_^PV^ is the photovoltaic gap determined from the derivatives of EQE_PV_ [25]. Based on the *^h^E*_CT_ position obtained through EL_B_ deconvolution, it required as much reorganization energy (*λ*) to match the onset part of FTPS-EQE (Figure 3). The Δ*^h^E*_CT_ values obtained in this analysis were 0.030 eV for PM6:Y6, 0.058 eV for PBDTTPD-HT:Y6, and 0.044 eV for PTB7-Th:Y6. In many previous studies, *E*_CT_ determination was performed using the maximum peak of EL_B_ [26,27,28]. However, as confirmed in the previous discussion, the maximum peak region of the EL_B_ contains a lot of the singlet contribution of the acceptor. Although a Gaussian fit can be matched to the FTPS-EQE spectrum, even if the CT state is analyzed based on the maximum of EL_B_, the extracted CT state value was nearly the same as or even larger than the bandgap of the Y6 acceptor (see Figure S5 and Table S3). Furthermore, many researchers simply considered *E*_CT_ to be the same as *qV*_OC_ in the Shockley–Queisser (SQ) limit (*qV*_OC_^SQ^). However, these two values have different theoretical backgrounds [29,30,31,32]. In our study, we compared the *qV*_OC_^SQ^ values obtained through energy loss analysis with *^h^E*_CT_ (Figure S6). The results clearly showed that *^h^E*_CT_ and *qV*_OC_^SQ^ have different values (Table S4). The results of the total energy loss analysis indicate that PM6:Y6 forms a large *V*_OC_ compared to PBDTTPD-HT:Y6, which has similar energy band configuration due to the low Δ*^h^E*_CT_, radiative energy loss (Δ*E*_2_ = *qV*_OC_^SQ^ − *qV*_OC_^rad^, where *qV*_OC_^rad^ is *qV*_OC_ in the radiative limit), and non-radiative energy loss (Δ*E*_3_ = *qV*_OC_^rad^ − *qV*_OC_^nonrad^, where *qV*_OC_^nonrad^ is *qV*_OC_ in the non-radiative limit; details are presented in Appendix B). In the case of PTB7-Th:Y6, although hole transfer is slightly better than the PBDTTPD-HT:Y6 due to the lower Δ*^h^E*_CT_, PTB7-Th:Y6 has a large *V*_OC_ loss due to relatively large values of Δ*E*_2_ and Δ*E*_3_.

In general, two methods are mainly utilized to determine Δ*E*_3_. When *qV*_OC_^SQ^ and *qV*_OC_^rad^ are derived theoretically based on measured *J*_SC_ and FTPS-EQE, Δ*E*_3_ is determined by simply subtracting the *qV*_OC_^rad^ from the *V*_OC_ of the solar cells. Alternatively, Δ*E*_3_ can also be calculated using the theoretical approach given by Δ*E*_3_ = −*k*_B_*T*ln(EQE_EL_), where *k*_B_ is the Boltzmann constant, *T* is temperature, and EQE_EL_ is the external radiative efficiency. Considering that the EL_B_ of the nf-BHJ system consists of EL from the acceptor’s single recombination and EL from *^h^E*_CT_, once EQE_EL_ obtained based on EL_B_ is used, both the contribution of the acceptor and the contribution of *^h^E*_CT_ are already included in the Δ*E*_3_ calculation. The fact that the non-radiative voltage loss can be further split into these two contributions was already recognized and reported in previous studies [20]. However, the problem is to accurately extract the contribution of the acceptor from only EL_B_. It is expected to be very difficult to extract only the contribution of acceptors in a general situation where EL_A_ and *^h^E*_CT_ bands overlap each other. In particular, since there is also a degeneracy part between the *^h^E*_CT_ band and acceptor HOMO, it is currently impossible to completely separate the two components. Therefore, to obtain the exact Δ*^h^E*_CT_, the application of Marcus theory through our EL deconvolution is the most accurate method so far. The effect of *^h^E*_CT_ on energy loss is already included in recombination loss (Δ*E*_3_) if EL_B_ is used to extract EQE_EL_. On the other hand, the effect of *^e^E*_CT_ on energy loss is mainly reflected in Δ*E*_1_. ΔE_1_ should be selected with the larger of *^e^E*_CT_ and *qV*_OC_^SQ^ (see Figure 1a). Of course, since there is a possibility of recombination between an electron at the *^e^E*_CT_ level and a hole at the HOMO of the donor, the effect of *^e^E*_CT_ is also included in Δ*E*_3_ if EL_B_ is used to extract EQE_EL_.

### 3.2. IDIC-Acceptor-Based nf-BHJ Systems (Larger HOMO Offset)

**Figure 4:**
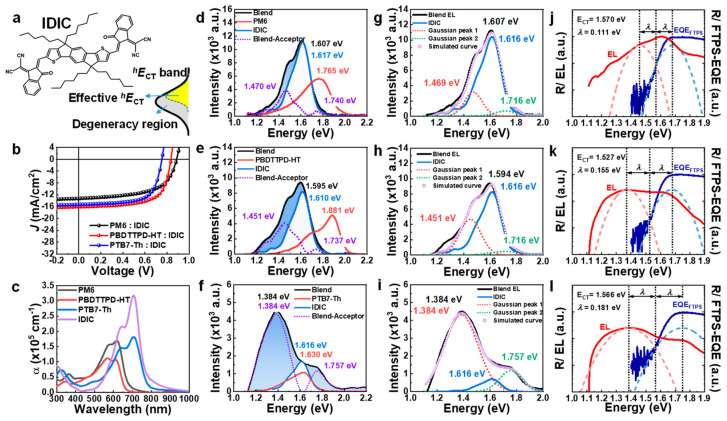
EL deconvolution analysis of the BHJ systems based on the IDIC acceptor. (**a**) Chemical structure of IDIC acceptor. (**b**) *J-V* characteristics of OPVs fabricated using IDIC acceptor. (**c**) Absorption coefficient spectra of PM6, PBDTTPD-HT, PTB7-Th, and IDIC. (**d**–**f**) EL spectrum of blend, donor, and acceptor; the difference in EL spectrum (shaded blue area) represents the EL contribution by *^h^E*_CT_. (**g**–**i**) Simulation of EL spectrum using Gaussian function and EL_A_. (**j**–**l**) Δ*^h^E*_CT_ determination based on the Marcus theory. The CT absorption (blue dotted line) and CT emission (red dotted line) were fitted using Gaussian function.

Next, we applied the EL deconvolution method to another NFA system, a BHJ blend with IDIC. The chemical structure and *J*-*V* characteristics of the IDIC system are shown in Figure 4a,b. The HOMO level of IDIC is similar to that of Y6, but it has a larger bandgap. Therefore, the absorption of IDIC largely overlaps with the donor polymers (Figure 4c). In particular, the absorption of IDIC completely overlaps with PTB7-Th absorption. These characteristics are directly reflected in the composition of EQE (Figure S7). For PM6:IDIC and PBDTTPD-HT:IDIC, though, the onset of EQE_PV_ is based on the photo-carriers created by the absorption of the acceptors. In the case of the PTB7-Th:IDIC, both donor and acceptor appear to be involved. Figure 4d–f shows the difference between EL_B_ and EL_A_. The peculiar point is that the contribution of the effective *^h^E*_CT_ was symmetric with respect to the HOMO of the acceptor in the case of Y6, whereas the IDIC system has an asymmetric shape where the lower-energy side is dominant. Thus, the effective *^h^E*_CT_ region is formed only on the upper side of the CT-band. In the case of PM6:IDIC and PBDTTPD-HT:IDIC, the maximum peak of EL_B_ was the same as that of EL_A_, but in the case of PTB7-Th:IDIC, the maximum part of EL_B_ was completely different from that of EL_A_. It can be seen that the CT-band contribution is much larger in PTB7-Th:IDIC only by a simple comparison of these EL deconvolutions. With this simple EL deconvolution analysis, it can be simply confirmed that the *^h^E*_CT_ band contribution in PTB7-Th:IDIC is much larger compared to PM6:IDIC and PBDTTPD-HT:IDIC. Exact Δ*^h^E*_CT_ obtained through accurate Gaussian fitting (Figure 4g–i) and Marcus theory (Figure 4j–l) for PM6:IDIC, PBDTTPD-HT:IDIC are shown in Table 1. The overall energy loss analysis results for the IDIC system are summarized in Figure S8 and Table S5. Note that it is impossible to distinguish whether it is Δ*^h^E*_CT_ or Δ*^e^E*_CT_ only with the current method in the case of PTB7-Th:IDIC blend. Of course, this discernment is also not possible with the conventional method (Gaussian fitting of FTPS-EQE and then matching to the maximum of EL_B_). The conventional method also gave unacceptable values (Figure S9 and Table S6).

### 3.3. IEICO-4F-Acceptor-Based BHJ Systems (Smaller HOMO Offset)

**Figure 5:**
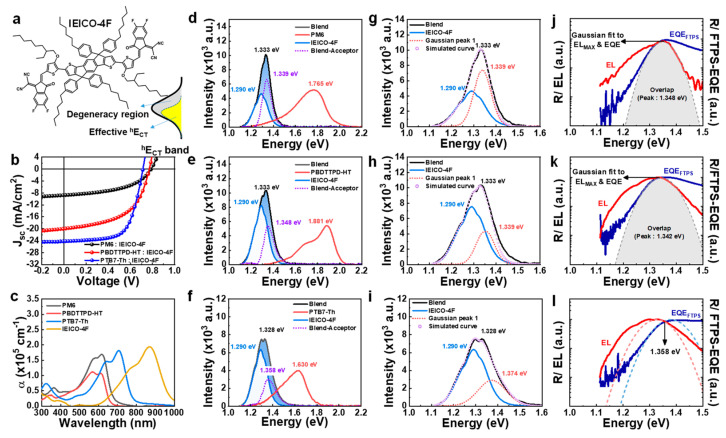
EL deconvolution analysis of the BHJ systems based on the IEICO-4F acceptor. (**a**) Chemical structure of IEICO-4F acceptor. (**b**) *J-V* characteristics of BHJ OPVs fabricated with IEICO-4F acceptor. (**c**) Absorption coefficient spectra of PM6, PBDTTPD-HT, PTB7-Th, and IEICO-4F. (**d**–**f**) EL spectrum of blend, donor, acceptor, and the difference in EL spectrum. (**g**–**i**) Simulation of EL spectrum using Gaussian function and EL_A_. One Gaussian fitting was obtained from EL_B_-EL_A_. (**j**–**l**) Δ*^h^E*_CT_ determination based on the Marcus theory. The CT absorption (blue dotted line) and CT emission (red dotted line) were fitted using Gaussian function, but indistinguishable from singlet band.

What if the acceptor’s HOMO is lower than the donor’s HOMO while having a low bandgap? We selected IEICO-4F as a material that satisfies these conditions (Supplementary Figure S3). The chemical structures and *J*-*V* characteristics of the IEICO-4F system are shown in Figure 5a,b. Since IEICO-4F has a lower bandgap than Y6, there is no overlap with that of absorption of the donor like Y6 (Figure 5c), and the EL_B_ and EL_D_ are also separated. In the IEICO-4F system, the onset of EQE is formed by only the contribution from the acceptor. Figure 5d–f shows the difference between EL_B_ and EL_A_. In the case of IEICO-4F, unlike IDIC, the difference between EL_B_ and EL_A_ does not appear at low energy but is formed in a higher-energy region (Figure 5d–f). The low-energy part of EL_B_ exactly overlaps with the EL_A_. Thus, the effective *^h^E*_CT_ region is formed only on the lower side of the CT band. The EL_B_ is perfectly simulated with the EL_A_ spectrum and one additional Gaussian (Figure 5g–i). The absence of the low-energy CT-band leads to a complete overlap of EQE fitting and EL fitting in Marcus theory analysis for PM6:IEICO-4F and PBDTTPD-HT:IEICO-4F, as shown in Figure 5j,k. The lower effective *^h^E*_CT_ than the HOMO of the acceptor indicates an insufficient HOMO offset for charge separation. The low *J*_SC_ in PM6:IEICO-4F and PBDTTPD-HT:IEICO-4F are attributed to this charge-separation problem. Only PTB7-Th:IEICO-4F has a proper condition for *^h^E*_CT_ formation. As a result, PTB7-Th:IEICO-4F shows the best photovoltaic performance. In fact, the values of *J*_SC_ are quite predictable by simple comparison of the lower effective *^h^E*_CT_ size. For PM6:IEICO-4F, which has a relatively large size of lower effective *^h^E*_CT_, the smallest *J*_SC_ was shown, and in PTB7-Th:IEICO-4F, which has a relatively small size of lower effective *^h^E*_CT_, the largest *J*_SC_ was formed. Sufficient hole transfer seems to be made through the degeneracy region. It is possible that in nf-BHJ, a certain level of hole offset may not be a prerequisite. Lastly, the overall energy loss composition of PTB7-Th:IEICO-4F was similar to that of PM6:Y6. Nevertheless, the lower photovoltaic performance of PTB7-Th:IEICO-4F relative to that of PM6:Y6 is attributed to the fact that the absorption coefficient of IEICO-4F itself is lower than that of Y6.

### 3.4. Hole Decay and Transfer Time at ^h^E_CT_

**Figure 6:**
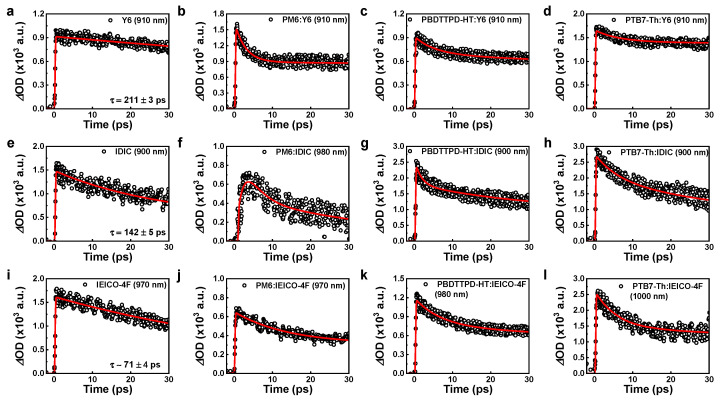
The decay profiling of hole-transfer time obtained from the TA measurements. (**a**) Pristine Y6, (**b**) PM6:Y6, (**c**) PBDTTPD-HT:Y6, (**d**) PTB7-Th:Y6, (**e**) pristine IDIC, (**f**) PM6: IDIC, (**g**) PBDTTPD-HT: IDIC, (**h**) PTB7-Th: IDIC, (**i**) pristine IEICO-4F, (**j**) PM6: IEICO-4F, (**k**) PBDTTPD-HT: IEICO-4F, (**l**) PTB7-Th: IEICO-4F.

The effect of the configuration change of the hole-transfer state on the hole-transfer time was investigated through transient absorption (TA) measurement. Figure 6 shows the decay profiling of the hole-transfer time obtained from the TA measurements for acceptors and BHJ films used in this study. Detailed values of the hole-transfer time and PL-quenching efficiency are listed in Table 2. In the case of the BHJ system with a Y6 acceptor, the measured hole-transfer time was much faster than the TA decay time of the Y6 film (~211 ps). The hole-transfer time was the fastest in PM6:Y6 (~2.8 ps), became slower in PBDTTPD-HT:Y6 (~5.1 ps), and showed the lowest value in PTB7-Th:Y6 (~7.0 ps). This order of hole-transfer time is well correlated with the order of the *^h^E*_CT_ area size obtained in EL deconvolution (see Figure 2h–j). The fast hole-transfer property delivers a lower recombination rate. Thus, the PL-quenching efficiency was the highest in PM6:Y6 (93%), and it was the lowest in PTB7-Th:Y6 (78%), which has the slowest hole-transfer rate (Figure S18). In the BHJ system with the IDIC acceptor (Figure 6e–h), the PBDTTPD-HT:IDIC exhibited the fastest hole-transfer time of ~1.8 ps, even faster than PM6:Y6. This is probably because the upper *^h^E*_CT_ region acting as an obstruction region of hole transfer is smaller. However, the overall PCE was lower than that of Y6-based devices. This is because of the decreased *J*_SC_ due to the large bandgap characteristics of IDIC. The longest hole-transfer time and the lowest PL-quenching efficiency were observed in PTB7-Th:IDIC, with the largest upper *^h^E*_CT_ region. For the BHJ systems with IEICO-4F, a prolonged hole-transfer time of ~13.0 ps was observed for PM6:IEICO-4F, and even the hole-transfer time of pristine IEICO-4F was remarkably faster (~71 ps) than other acceptors (Figure 6i). This is because of the relatively large upper *^h^E*_CT_ and the small degeneracy region, as predicted by the EL deconvolution result. Since most of the hole-transfer levels were created on the upper *^h^E*_CT_ region that causes hole-transfer retardation, the hole-transfer characteristics were significantly degraded compared to other acceptor systems. However, PTB7-Th: IEICO-4F, with a relatively small upper *^h^E*_CT_ region, showed a fast hole-transfer time and high hole-quenching efficiency. Thereby, PTB7-Th: IEICO-4F exhibited relatively higher performance within BHJ systems based on the IEICO-4F acceptor. 

## 4. Conclusions

In summary, we have presented a more accurate analysis method that can obtain the exact value of Δ*^h^E*_CT_ in NFA-based OPV by extracting information on *^h^E*_CT_ through EL_B_ deconvolution. We have applied our EL_B_ deconvolution method on nine BHJ systems consisting of a combination of three donor polymers (PM6, PBDTTPD-HT, PTB7-Th) and three NFAs (Y6, IDIC, IEICO-4F). To extract the exact Δ*^h^E*_CT_, the following conditions must be satisfied to apply our EL_B_ deconvolution method. (i) Absorption of the donor and acceptor must be separated sufficiently. Accordingly, (ii) the composition of EQE should be clearly divided into donor and acceptor contribution parts. In particular, the initial EQE_PV_ onset of an OPV with NFA should be formed only by the contribution of the acceptor. If these conditions are satisfied, through EL_B_ deconvolution, it is possible to specify exactly which part of the broad EL_B_ should be used to apply the Marcus theory, and based on the information on *^h^E*_CT_, we were able to obtain a fairly accurate Δ*^h^E*_CT_. Accurate determination of *^h^E*_CT_ is very important to understand the overall charge dynamics of NFAs-based OPVs. Since improved performance and stability are required for the commercialization of OPVs, it is expected that our method can be implemented for the development of better photoactive layer materials with improved performance in the future.

## Figures and Tables

**Table 1:** Extracted *^h^E*_CT_ values of each BHJ system obtained using EL_B_ deconvolution method.

BHJ	*E*_g_	*^h^E*_CT_	Δ*^h^E*_CT_
PM6: Y6	1.418	1.388	0.030
PBDTTPD-HT: Y6	1.431	1.373	0.058
PTB7-Th: Y6	1.401	1.357	0.044
PM6: IDIC	1.601	1.570	0.031
PBDTTPD-HT: IDIC	1.573	1.527	0.046
PTB7-Th: IDIC	1.611	1.566	0.045
PM6: IEICO-4F	1.323	n/a	n/a
PBDTTPD-HT: IEICO-4F	1.333	n/a	n/a
PTB7-Th: IEICO-4F	1.330	1.358	n/a (−0.028)

Unit: eV.

**Table 2:** Hole-transfer time and PL-quenching efficiency of BHJ used in this study.

Blend	Hole-Transfer Time (ps)	$\eta_{\mathrm{PL}}$* (%)
PM6:Y6	2.8 ± 0.3	93
PBDTTPD-HT:Y6	5.1 ± 0.5	81
PTB7-Th:Y6	7.0 ± 0.4	78
PM6:IDIC	2.4 ± 0.7	75
PBDTTPD-HT:IDIC	1.8 ± 0.4	81
PTB7-Th:IDIC	6.0 ± 0.4	54
PM6:IEICO-4F	13.0 ± 1.7	72
PBDTTPD-HT:IEICO-4F	7.8 ± 0.7	83
PTB7-Th:IEICO-4F	5.2 ± 0.8	96

*$\eta_{\mathrm{PL}}$: PL-quenching efficiency.

## Data Availability

The data presented in this study are available on request from the corresponding author.

## Supplementary Materials

The following supporting information can be downloaded at: https://www.mdpi.com/article/10.3390/polym15204042/s1, Figure S1: *E*_CT_ determination of PM6:PC_71_BM based on the Marcus theory; Figure S2: Chemical structures of polymer donors used in this study; Figure S3: Energy-level diagram of polymer donors and non-fullerene acceptors; Figure S4: EQE_PV_ spectra of donor, acceptor, and BHJs where the acceptor is Y6; Figure S5: Example of incorrect *E*_CT_ determination using the maximum peak of EL_B_; Figure S6: Energy-loss analysis of BHJs with Y6 based on the detailed balance and reciprocity theorem; Figure S7: EQE_PV_ spectra of donor, acceptor, and BHJs where the acceptor is IDIC; Figure S8: Energy-loss analysis of BHJs with IDIC based on the detailed balance and reciprocity theorem; Figure S9: Example of incorrect E_CT_ determination using the maximum peak of EL_B_; Figure S10: EQE_PV_ spectra of donor, acceptor, and BHJs where the acceptor is IEICO-4F; Figure S11: Energy-loss analysis of BHJs with IEICO-4F based on the detailed balance and reciprocity theorem; Figure S12: Absorption and PL spectra of polymer donors and non-fullerene acceptors; Figure S13: Absorption coefficient spectra of BHJ systems; Figure S14: Bandgap distribution of BHJs obtained from the derivatives of EQE_PV_; Figure S15: Transient absorption spectra of Y6 and Y6-based BHJ films; Figure S16: Transient absorption spectra of IDIC and IDIC-based BHJ films; Figure S17: Transient absorption spectra of IEICO-4F and IEICO-4F-based BHJ films; Figure S18: PL spectra of acceptor and BHJ films used in this study; Table S1: *E*_CT_ values of PM6:PC_71_BM where the Marcus theory is applied to CT feature at the onset of FTPS-EQE (A) or maximum peak of FTPS-EQE (B) in Figure S1; Table S2: Photovoltaic performance of BHJs used in this study; Table S3: Example of incorrection Δ*E*_CT_ determination of BHJs with Y6; Table S4: Energy-loss results of BHJs with Y6; Table S5: Energy-loss results of BHJs with IDIC; Table S6: Example of incorrect energy-loss results of BHJs with IDIC; Table S7: Energy-loss results of BHJs with IEICO-4F.

## Appendix A

### Marcus Theory

According to the Frank–Condon principle, the absorption and emission have to be exactly symmetric for the lowest vibronic state (cross point) because the electronic transitions are swift compared with nuclear motions. Similarly, this can be determined by the Marcus theory, which explains the rates of electron transfer reactions, and the spectral regions of CT absorption and emission are expressed as follows [32]:

(A1) $$\mathrm{EQE}(E)\cdot E=\frac{\alpha_{\mathrm{absorption}}}{\sqrt{4\pi \lambda k_{B}T}}\exp\left[-\frac{{\left(E_{\mathrm{CT}}+\lambda -E\right)}^{2}}{4\lambda k_{B}T}\right]$$

(A2) IfE=αemission4πλkBTexp−ECT−λ−E24λkBT

where α is the coupling constant, λ is the reorganization energy, $k_{B}$ is Boltzmann’s constant, *T* is temperature, *I_f_* is the emission rate, and *E* is the corresponding energy to the absorption/emission spectrum.

## Appendix B

### Appendix B.1. Energy Loss Model

The energy loss based on the detailed balance and reciprocity can be expressed as the sum of three components, as follows [31]:

(A3) $$E_{\mathrm{loss}}={\Delta E}_{1}+{\Delta E}_{2}+{\Delta E}_{3}\;=\left(E_{g}-qV_{\mathrm{OC}}^{\mathrm{SQ}}\right)+\left({\mathrm{qV}}_{\mathrm{OC}}^{\mathrm{SQ}}-{\mathrm{qV}}_{\mathrm{OC}}^{\mathrm{rad}}\right)+({\mathrm{qV}}_{\mathrm{OC}}^{\mathrm{rad}}-{\mathrm{qV}}_{\mathrm{OC}})$$

where ${\mathrm{qV}}_{\mathrm{OC}}^{\mathrm{rad}}$ is radiative limited ${\mathrm{qV}}_{\mathrm{OC}}$ in consideration of EQE, and $V_{\mathrm{OC}}$ is open-circuit voltage measured from the current density–voltage (*J-V*) curve. ${\Delta E}_{1}$ is voltage loss due to mismatches in the angle between incident sunlight and emission and carrier concentration between generation and the radiation from the cell. ${\Delta E}_{2}$ is the radiative voltage loss due to the imperfection of EQE since $qV_{\mathrm{OC}}^{\mathrm{SQ}}$ considers the assumption of 100% of EQE, and ${\Delta E}_{3}$ is non-radiative voltage loss due to the non-radiative recombination.

#### Radiative Limit Open-Circuit Voltage

Referring to the reciprocity theorem [33], ${\mathrm{qV}}_{\mathrm{OC}}^{\mathrm{rad}}$ states the radiative limit including the EQE of solar cells:

(A4) $${\mathrm{qV}}_{\mathrm{OC}}^{\mathrm{rad}}=\frac{k_{B}T}{q}ln\left[\frac{J_{\mathrm{sc}}}{J_{0,\mathrm{rad}}}+1\right]=\frac{k_{B}T}{q}ln\left[\frac{q\int_{0}^{\infty }\Omega_{\mathrm{PV}}\left(E\right)\phi_{\mathrm{sun}}\left(E\right)dE}{q\int_{0}^{\infty }\Omega_{\mathrm{PV}}\left(E\right)\phi_{\mathrm{BB}}\left(E\right)dE}+1\right].$$

where

(A5) $$\phi_{\mathrm{BB}}\left(E\right)=\frac{2\pi E^{2}}{h^{3}c^{2}}\exp\left[-\frac{E}{k_{B}T}\right]$$

is the blackbody radiation from the semiconductor and $\Omega_{\mathrm{PV}}\left(E\right)$ is the EQE of the solar cell. Herein, $V_{\mathrm{oc}}^{\mathrm{rad}}$ must be the *V_OC_* of the solar cell in the situation that radiative recombination in the only process in the total recombination. Thus, the non-radiative energy loss is defined as the difference between $V_{\mathrm{oc}}^{\mathrm{rad}}$ and real *V_OC_*, as described in Equation (A3). By the definition of total energy loss, ${\Delta E}_{3}$ is the residual value of voltage losses above, i.e.,

(A6) $${\Delta E}_{3}={E_{\mathrm{loss}}-(\Delta E}_{1}+{\Delta E}_{2})={\mathrm{qV}}_{\mathrm{OC}}^{\mathrm{rad}}-{\mathrm{qV}}_{\mathrm{OC}}$$

Meanwhile, EQE_EL_ is defined as the radiative recombination current divided by the injected current for EL emission:

(A7) $${\mathrm{EQE}}_{\mathrm{EL}}=\frac{J_{\mathrm{emitted}}\;(V)}{J_{\mathrm{injected}}\;(V)}=\frac{J_{\mathrm{rad}}\;(V)}{J_{\mathrm{rad}}\left(V\right)+J_{\mathrm{non}-\mathrm{rad}}\;(V)}$$

Because EQE_EL_ refers how the radiative recombination possesses in total recombination, ${\Delta E}_{3}$ is proportional to -ln(EQE_EL_) [33]:

(A8) $${{\Delta E}_{3}=qV}_{\mathrm{OC}}^{\mathrm{rad}}-{\mathrm{qV}}_{\mathrm{OC}}≅-\frac{k_{B}T}{q}ln\left[{\mathrm{EQE}}_{\mathrm{EL}}\right]$$
