# Supplementary material for: Investigation of Hole-Transfer Dynamics through Simple EL De-Convolution in Non-Fullerene Organic Solar Cells
Source: Polymers (Basel). 2023 Oct 10;15(20):4042. doi: 10.3390/polym15204042 (PMC10610510; doi:10.3390/polym15204042)
Supplement: Supplementary file 1 [file polymers-15-04042-s001.zip › polymers-2624173-supplementary.pdf]

Supplementary Information

Investigation on hole transfer dynamics through simple EL de-convolution in non-fullerene organic solar cells

Dongchan Lee [1†], Do Hui Kim[1†], Chang-Mok Oh[3†], Sujung Park[1], Narra Vamsi Krishna[2], Febrian Tri Adhi Wibowo[2], In-Wook Hwang[3], Sung-Yeon Jang[2], and Shinuk Cho[1]*

[1]Department of Semiconductor Physics and Energy Harvest Storage Research Center, University of Ulsan; Ulsan 44610, Republic of Korea.

[2]Department of Energy Engineering and School of Energy and Chemical Engineering, Ulsan National Institute of Science and Technology (UNIST); Ulsan 44919, Republic of Korea.

[3]Advanced Photonics Research Institute, Gwangju Institute of Science and Technology, Gwangju 61005, Republic of Korea.

*Corresponding author. E-mail: sucho@ulsan.ac.kr (Shinuk Cho)

[†]These three authors (Dongchan Lee, Do Hui Kim, Chang-Mok Oh) contributed equally to this work.

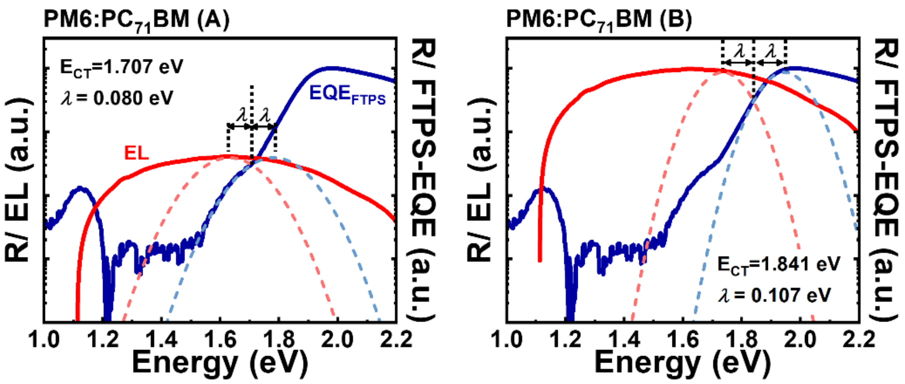

Figure S1. E_{CT} determination of PM6:PC$_{71}$BM based on the Marcus theory. The device based on fullerene acceptor clearly showed $^{e}E_{CT}$ feature on the onset of FTPS-EQE. (**A**) Correct Gaussian fitting for the $^{e}E_{CT}$ band. (**B**) Incorrect Gaussian fitting gives inaccurate $^{e}E_{CT}$ values.

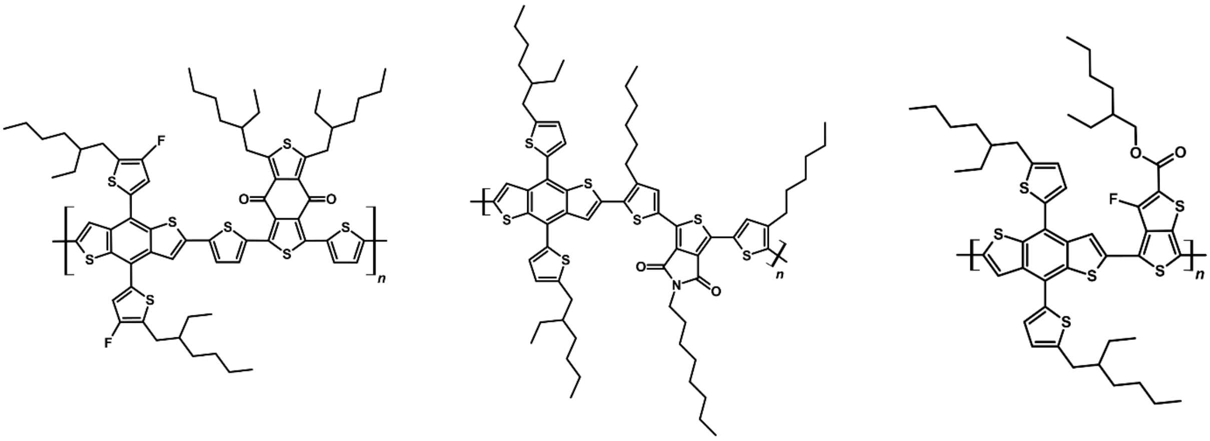

PM6 **PBDTTPD-HT** **PTB7-Th**

Figure S2. Chemical structures of polymer donors used in this study.

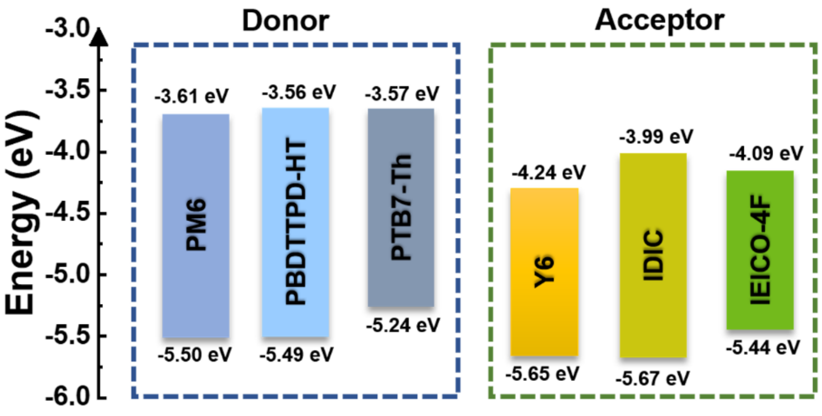

Figure S3. Energy level diagram of polymer donors and non-fullerene acceptors.

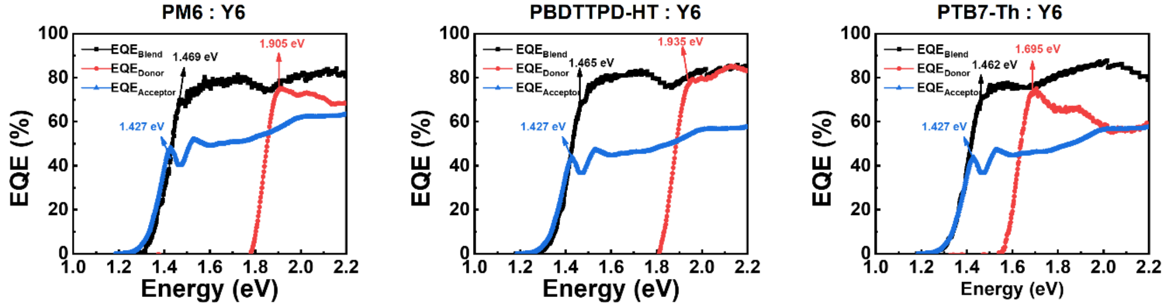

Figure S4. EQE_{PV} spectra of donor, acceptor, and BHJs where the acceptor is Y6. Onset of $EQE_{Acceptor}$ equals that of EQE_{Blend}. EQE_{Donor} does not contribute to low-energy region of the EQE_{Blend}, which means that only charges from the acceptor contribute to the onset part of the EQE_{Blend}.

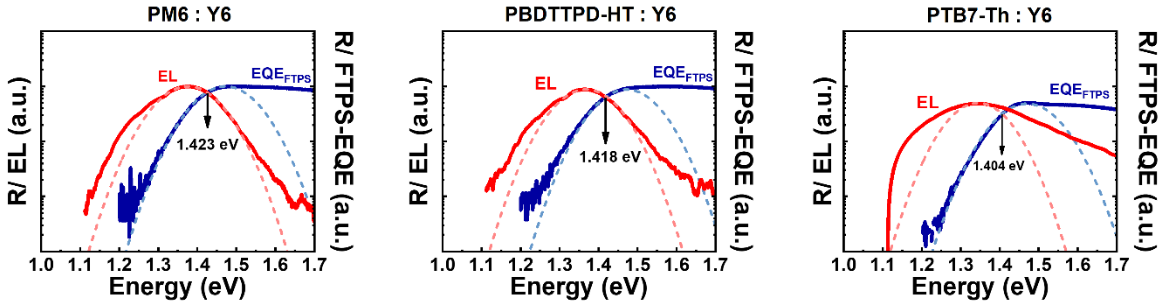

Figure S5. Example of incorrect E_{CT} determination using the maximum peak of EL_B. In the case of PM6 and PTB7-Th, the extracted E_{CT} level had a higher value than that of E_g.

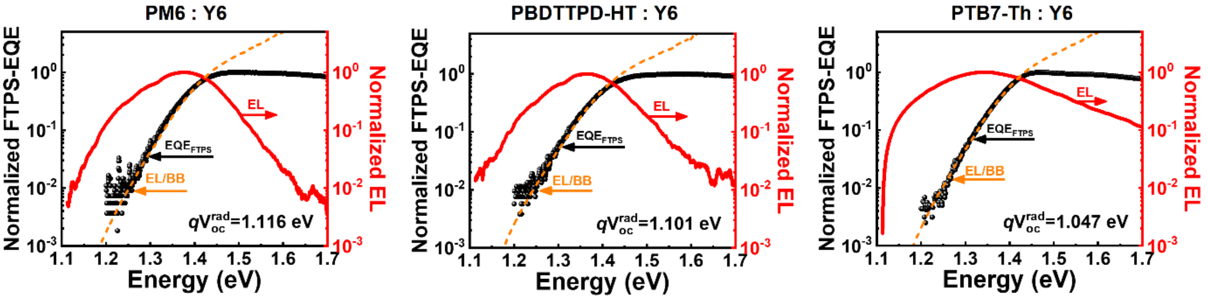

Figure S6. Energy loss analysis of BHJs with Y6 based on the detailed balance and reciprocity theorem. EL/BB was fit to onset of the FTPS-EQE to determine qV_{oc}^{rad}.

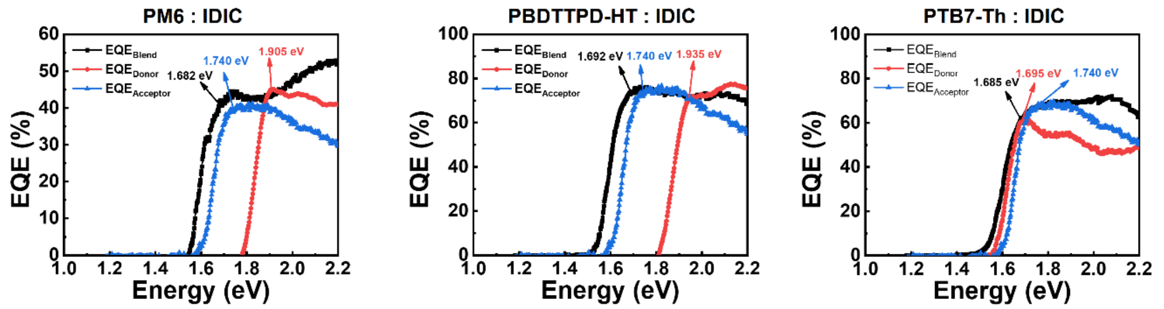

Figure S7. EQE_{PV} spectra of donor, acceptor, and BHJs where the acceptor is IDIC. Mismatch of EQE onset of blend and acceptor is attributed to larger CT band than in other BHJs (in particular, the onset of EQE_{Blend} is lower than that of $EQE_{Acceptor}$).

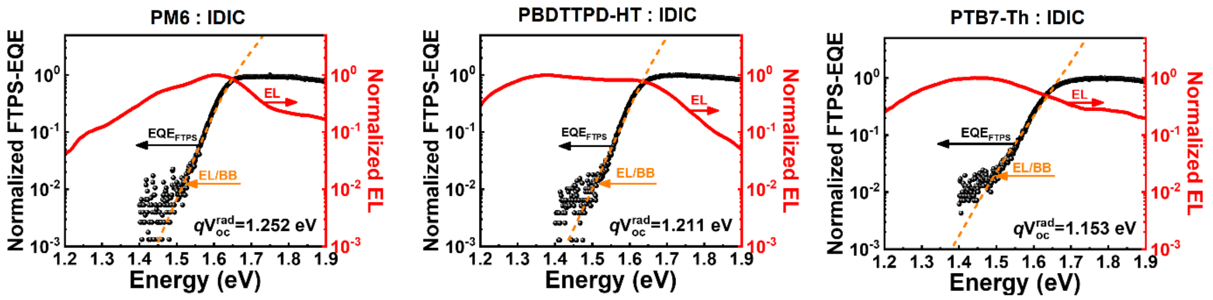

Figure S8. Energy loss analysis of BHJs with IDIC based on the detailed balance and reciprocity theorem. EL/BB was fit to onset of the FTPS-EQE to determine qV_{oc}^{rad}.

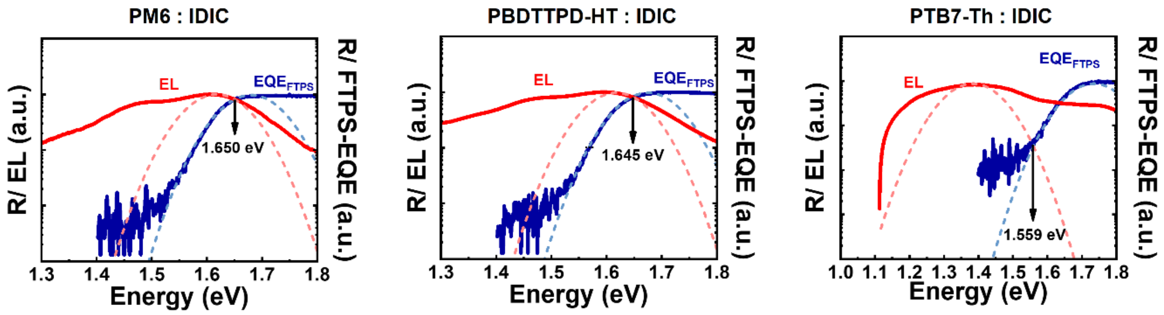

Figure S9. Example of incorrect E_{CT} determination using the maximum peak of EL_B.

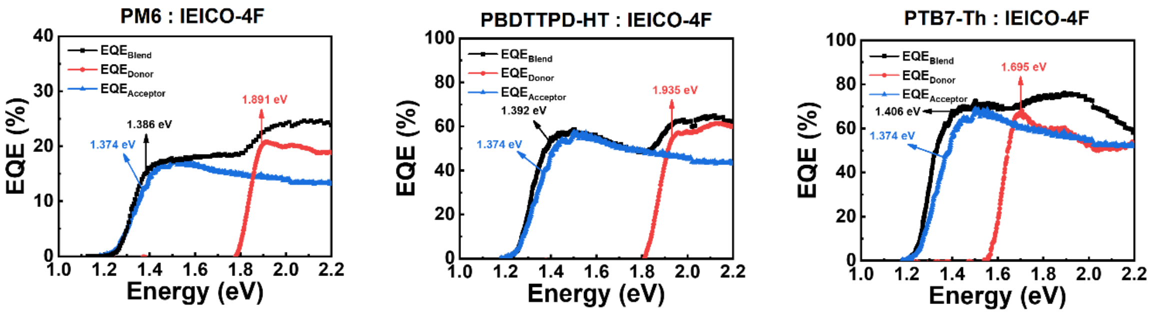

Figure S10. EQE_{PV} spectra of donor, acceptor, and BHJs where the acceptor is IEICO-4F. Each EQE behavior trend is similar to Y6 systems.

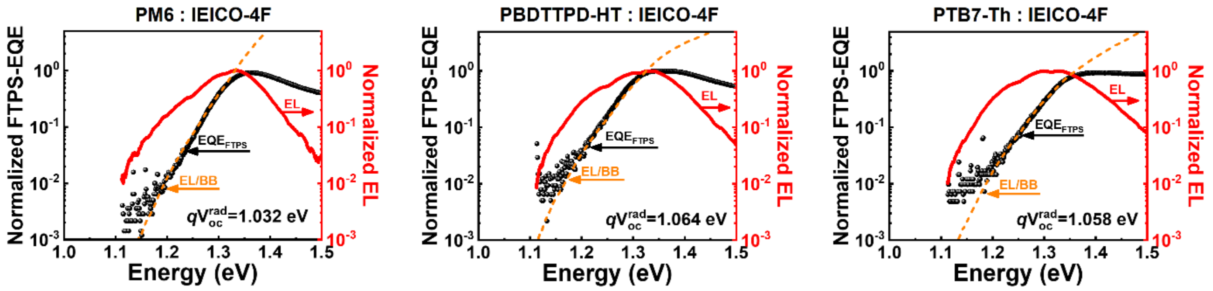

Figure S11. Energy loss analysis of BHJs with IEICO-4F based on the detailed balance and reciprocity theorem. EL/BB was fit to the onset of the FTPS-EQE to determine qV_{oc}^{rad}.

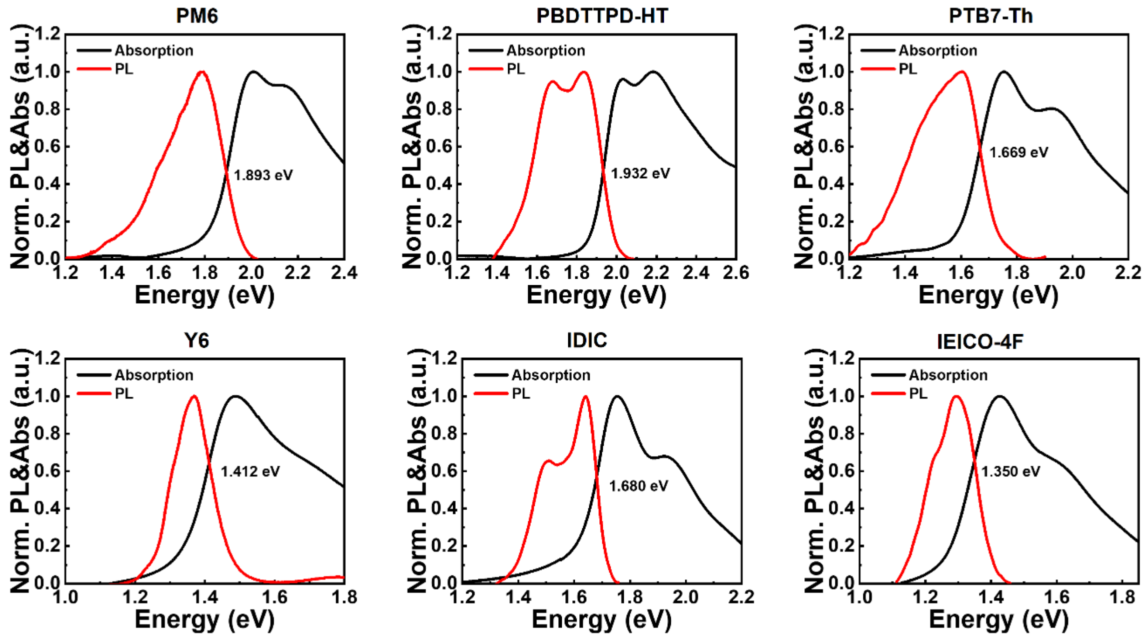

Figure S12. Absorption and photoluminescence (PL) spectra of polymer donors and non-fullerene acceptors. Crosspoint position of absorption and PL was estimated to singlet energy.

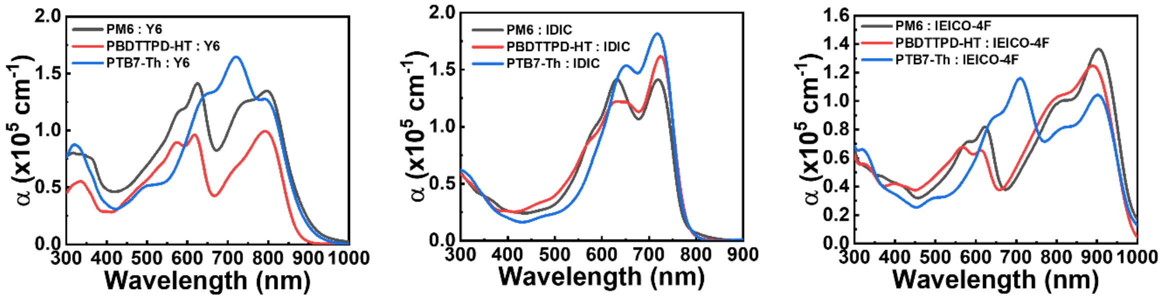

Figure S13. Absorption coefficient spectra of BHJ systems.

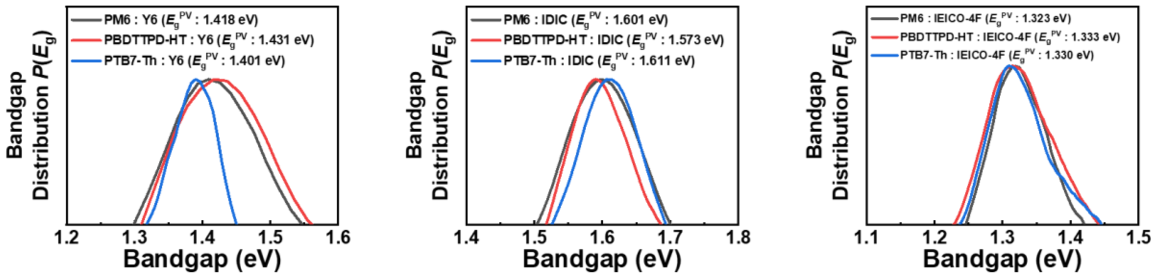

Figure S14. Bandgap distribution of BHJs obtained from the derivatives of the EQE_{PV}.

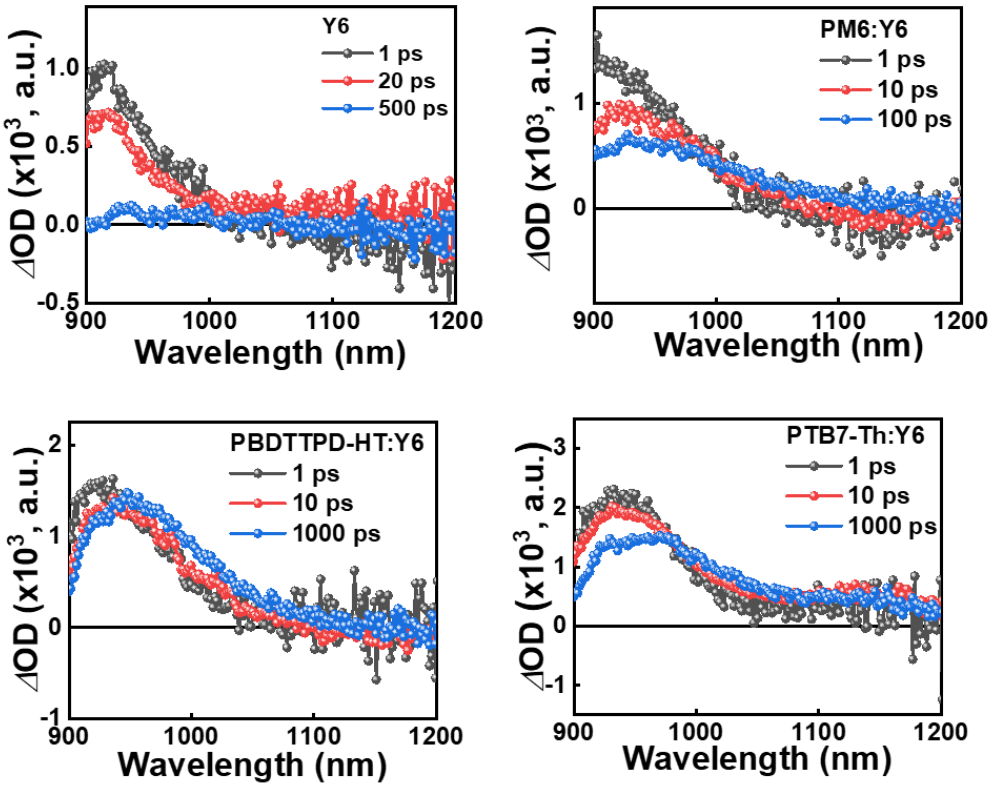

Figure S15. Transient absorption spectra of Y6 and Y6-based BHJ films.

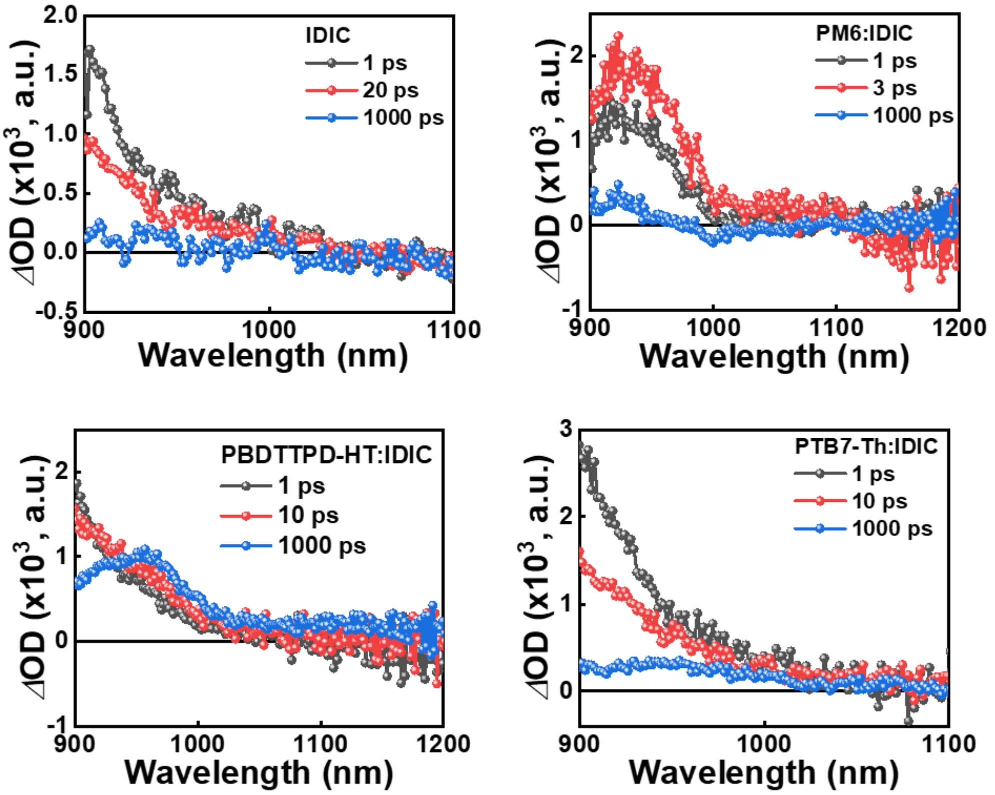

Figure S16. Transient absorption spectra of IDIC and IDIC-based BHJ films.

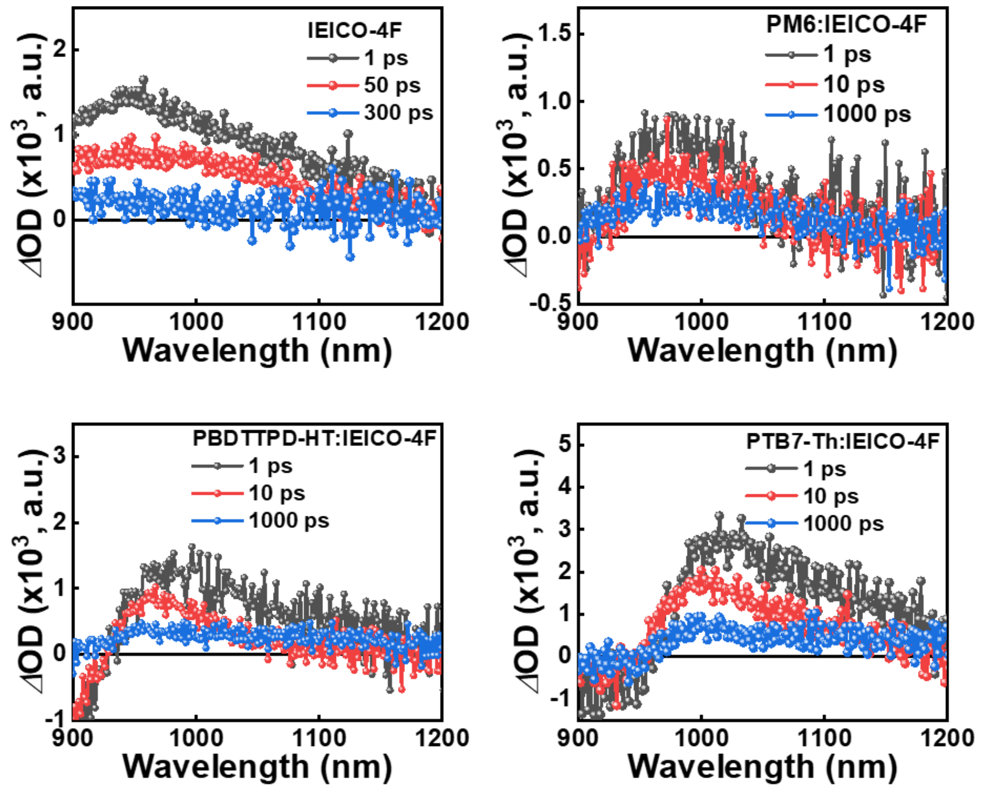

Figure S17. Transient absorption spectra of IEICO-4F and IEICO-4F-based BHJ films.

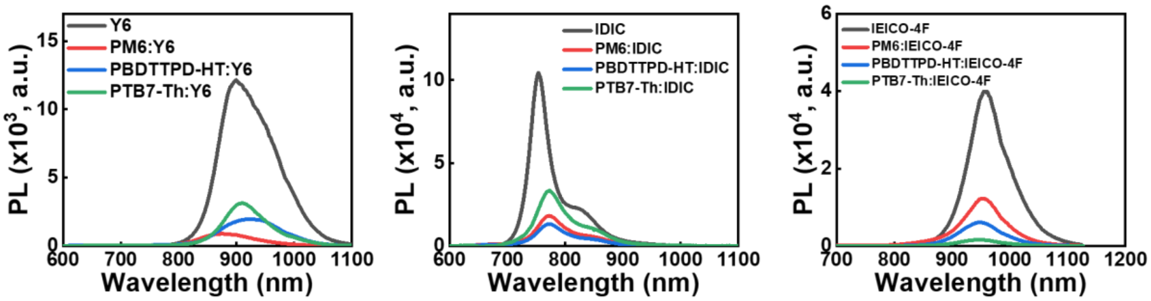

Figure S18. Photoluminescence spectra of acceptor and BHJ films used in this study.

Table S1. E_{CT} values of PM6:PC_{71}BM where the Marcus theory applied to CT feature at the onset of FTPS-EQE (A) or maximum peak of FTPS-EQE (B) from **Figure S1**.

	E_g (eV)	E_{CT} (eV)	ΔE_{CT} (eV)
**PM6:PC_{71}BM**	1.855	1.854	0.001
**PM6:PC_{71}BM (A)**	1.855	1.707	0.148
**PM6:PC_{71}BM (B)**	1.855	1.841	0.014

Table S2. Photovoltaic performance of BHJs used in this study.

**Blend**	J_{sc} (mA/cm^2)	V_{oc} (V)	**FF (%)**	**PCE (%)**
**PM6:Y6**	26.27	0.862	73.8	16.70
**PBDTTPD-HT : Y6**	27.07	0.773	63.8	13.35
**PTB7-Th:Y6**	23.52	0.672	69.9	11.04
**PM6:IDIC**	15.60	0.908	67.1	9.51
**PBDTTPD-HT : IDIC**	16.73	0.862	70.6	10.18
**PTB7-Th:IDIC**	15.28	0.753	67.4	7.76
**PM6 : IEICO-4F**	8.75	0.787	48.4	3.33
**PBDTTPD-HT : IEICO-4F**	19.99	0.770	53.4	8.22
**PTB7-Th : IEICO-4F**	24.19	0.713	65.0	11.21

Table S3. Example of incorrection ΔE_{CT} determination of BHJs with Y6. E_{CT} was determined where CT emission peak position is the maximum of EL_B.

Donor	E_g	E_{CT}	ΔE_{CT}
**PM6**	1.418	1.423	-0.005
**PBDTTPD-HT**	1.431	1.418	0.013
**PTB7-Th**	1.401	1.404	-0.003

Table S4. Energy loss results of BHJs with Y6. ${}^{h}E_{CT}$ was analyzed in Figure. 2 in the main text.

Donor	E_g	${}^{h}E_{CT}$	$\Delta^{h}E_{CT}$	qV_{OC}^{SQ}	ΔE_1	qV_{oc}^{rad}	ΔE_2	qV_{oc}	ΔE_3	Cal. E_3	EQE_{EL}
	(eV)										(%)
**PM6**	1.418	1.388	0.030	1.160	0.258	1.116	0.044	0.862	0.254	0.272	2.645×10^{-3}
**PBDTTPD-HT**	1.431	1.373	0.058	1.172	0.259	1.101	0.084	0.775	0.326	0.337	2.153×10^{-4}
**PTB7-Th**	1.401	1.357	0.044	1.144	0.257	1.047	0.097	0.672	0.375	0.361	8.656×10^{-5}

Table S5. Energy loss results of BHJs with IDIC. ${}^{h}E_{CT}$ was determined using the low energy side peak from deconvolution of EL_B.

Donor	E_g	${}^{h}E_{CT}$	$\Delta^{h}E_{CT}$	qV_{OC}^{SQ}	ΔE_1	qV_{oc}^{rad}	ΔE_2	qV_{oc}	ΔE_3	Cal. E_3	EQE_{EL}
	(eV)										(%)
**PM6**	1.601	1.570	0.031	1.331	0.270	1.252	0.079	0.908	0.344	0.339	1.198×10^{-4}
**PBDTTPD-HT**	1.573	1.527	0.046	1.305	0.268	1.211	0.094	0.856	0.355	0.372	5.686×10^{-5}
**PTB7-Th**	1.611	1.566	0.045	1.341	0.270	1.153	0.188	0.753	0.400	0.427	6.620×10^{-6}

Table S6. Example of incorrect energy loss results of BHJs with IDIC. In this analysis, E_{CT} was determined using

the maximum part of EL_B (Figure. S9).

Donor	E_g	$^hE_{CT}$	Δ^hE_{CT}	qV_{OC}^{SQ}	ΔE_1	qV_{oc}^{rad}	ΔE_2	qV_{oc}	ΔE_3	Cal. E_3	EQE_{EL}
	(eV)										(%)
**PM6**	1.601	1.650	-0.049	1.331	0.270	1.252	0.079	0.908	0.344	0.339	$1.198x10^{-4}$
**PBDTTPD-HT**	1.573	1.645	-0.072	1.305	0.268	1.211	0.094	0.856	0.355	0.372	$5.686x10^{-5}$
**PTB7-Th**	1.611	1.559	0.052	1.341	0.270	1.210	0.131	0.753	0.457	0.427	$6.620x10^{-6}$

Table S7. Energy loss results of BHJs with IEICO-4F. $^hE_{CT}$ could not be determined due to low HOMO offset between polymer donors and IEICO-4F acceptor.

Donor	E_g	$^hE_{CT}$	Δ^hE_{CT}	qV_{OC}^{SQ}	ΔE_1	qV_{oc}^{rad}	ΔE_2	qV_{oc}	ΔE_3	Cal. E_3	EQE_{EL}
	(eV)										(%)
**PM6**	1.323	x	x	1.071	0.252	1.032	0.039	0.787	0.245	0.272	$2.739x10^{-3}$
**PBDTTPD-HT**	1.333	x	x	1.080	0.253	1.064	0.016	0.770	0.294	0.282	$1.843x10^{-3}$
**PTB7-Th**	1.330	1.358	-0.028	1.077	0.253	1.058	0.019	0.704	0.373	0.354	$1.114x10^{-4}$
